# Genomic epidemiology and longitudinal sampling of ward wastewater environments and patients reveals complexity of the transmission dynamics of *bla*_KPC_-carbapenemase-producing Enterobacterales in a hospital setting

**DOI:** 10.1093/jacamr/dlae140

**Published:** 2024-09-03

**Authors:** N Stoesser, R George, Z Aiken, H T T Phan, S Lipworth, T P Quan, A J Mathers, N De Maio, A C Seale, D W Eyre, A Vaughan, J Swann, T E A Peto, D W Crook, J Cawthorne, A Dodgson, A S Walker, TRACE Investigators Group, Zoie Aiken, Oluwafemi Akinremi, Aiysha Ali, Julie Cawthorne, Paul Cleary, Derrick W Crook, Valerie Decraene, Andrew Dodgson, Michel Doumith, Matthew J Ellington, Ryan George, John Grimshaw, Malcolm Guiver, Robert Hill, Katie L Hopkins, Rachel Jones, Cheryl Lenney, Amy J Mathers, Ashley McEwan, Ginny Moore, Andrew Mumford, Mark Neilson, Sarah Neilson, Tim E A Peto, Hang T T Phan, Mark Regan, Anna C Seale, Nicole Stoesser, Jay Turner-Gardner, Vicky Watts, A Sarah Walker, Jimmy Walker, William Welfare, Neil Woodford, David H Wyllie

**Affiliations:** Nuffield Department of Medicine, University of Oxford, Oxford, UK; NIHR Health Protection Research Unit in Healthcare Associated Infections and Antimicrobial Resistance at University of Oxford in partnership with Public Health England, Nuffield Department of Medicine, Oxford, UK; NIHR Oxford Biomedical Research Centre, Oxford University Hospitals NHS Foundation Trust, John Radcliffe Hospital, Oxford, UK; Department of Microbiology, Manchester University NHS Foundation Trust, Manchester, UK; Department of Microbiology, Manchester University NHS Foundation Trust, Manchester, UK; Academic Unit of Clinical and Experimental Sciences, University of Southampton, Southampton, UK; Nuffield Department of Medicine, University of Oxford, Oxford, UK; Nuffield Department of Medicine, University of Oxford, Oxford, UK; NIHR Health Protection Research Unit in Healthcare Associated Infections and Antimicrobial Resistance at University of Oxford in partnership with Public Health England, Nuffield Department of Medicine, Oxford, UK; Department of Pathology, University of Virginia School of Medicine, Charlottesville, VA, USA; Goldman Group, EMBL-European Bioinformatics Institute, Cambridge, UK; Warwick Medical School - Health Sciences, University of Warwick, Coventry, UK; Nuffield Department of Medicine, University of Oxford, Oxford, UK; NIHR Health Protection Research Unit in Healthcare Associated Infections and Antimicrobial Resistance at University of Oxford in partnership with Public Health England, Nuffield Department of Medicine, Oxford, UK; NIHR Oxford Biomedical Research Centre, Oxford University Hospitals NHS Foundation Trust, John Radcliffe Hospital, Oxford, UK; Nuffield Department of Medicine, University of Oxford, Oxford, UK; Nuffield Department of Medicine, University of Oxford, Oxford, UK; Nuffield Department of Medicine, University of Oxford, Oxford, UK; NIHR Oxford Biomedical Research Centre, Oxford University Hospitals NHS Foundation Trust, John Radcliffe Hospital, Oxford, UK; Nuffield Department of Medicine, University of Oxford, Oxford, UK; NIHR Health Protection Research Unit in Healthcare Associated Infections and Antimicrobial Resistance at University of Oxford in partnership with Public Health England, Nuffield Department of Medicine, Oxford, UK; NIHR Oxford Biomedical Research Centre, Oxford University Hospitals NHS Foundation Trust, John Radcliffe Hospital, Oxford, UK; Department of Microbiology, Manchester University NHS Foundation Trust, Manchester, UK; Department of Microbiology, Manchester University NHS Foundation Trust, Manchester, UK; Nuffield Department of Medicine, University of Oxford, Oxford, UK; NIHR Health Protection Research Unit in Healthcare Associated Infections and Antimicrobial Resistance at University of Oxford in partnership with Public Health England, Nuffield Department of Medicine, Oxford, UK; NIHR Oxford Biomedical Research Centre, Oxford University Hospitals NHS Foundation Trust, John Radcliffe Hospital, Oxford, UK

## Abstract

**Background:**

Healthcare-associated wastewater and asymptomatic patient reservoirs colonized by carbapenemase-producing Enterobacterales (CPE) contribute to nosocomial CPE dissemination, but the characteristics and dynamics of this remain unclear.

**Methods:**

We systematically sampled wastewater sites (*n* = 4488 samples; 349 sites) and patients (*n* = 1247) across six wards over 6–12 months to understand bla_KPC_-associated CPE (KPC-E) diversity within these reservoirs and transmission in a healthcare setting. Up to five KPC-E-positive isolates per sample were sequenced (Illumina). Recombination-adjusted phylogenies were used to define genetically related strains; assembly and mapping-based approaches were used to characterize antimicrobial resistance genes, insertion sequences (ISs) and Tn*4401* types/target site sequences. The accessory genome was evaluated in some of the largest clusters, and those crossing reservoirs.

**Results:**

Wastewater site KPC-E-positivity was substantial [101/349 sites (28.9%); 228/5601 (4.1%) patients cultured]. Thirteen KPC-E species and 109 strains were identified using genomics, and 24% of wastewater and 26% of patient KPC-E-positive samples harboured one or more strains. Most diversity was explained by the individual niche, suggesting localized factors are important in selection and spread. Tn*4401* + flanking target site sequence diversity was greater in wastewater sites (*P* < 0.001), which might favour Tn*4401*-associated transposition/evolution. Shower/bath- and sluice/mop-associated sites were more likely to be KPC-E-positive (adjusted OR = 2.69; 95% CI: 1.44–5.01; *P* = 0.0019; and adjusted OR = 2.60; 95% CI: 1.04–6.52; *P* = 0.0410, respectively). Different strains had different bla_KPC_ dissemination dynamics.

**Conclusions:**

We identified substantial and diverse KPC-E colonization of wastewater sites and patients in this hospital setting. Reservoir and niche-specific factors (e.g. microbial interactions, selection pressures), and different strains and mobile genetic elements likely affect transmission dynamics. This should be considered in surveillance and control strategies.

## Introduction

Carbapenemase-producing Enterobacterales (CPE) are a global health threat,^1^ and treatment of CPE infections remains difficult. Major global carbapenemases include the MBLs (bla_NDM_, bla_VIM_, bla_IMP_), some oxacillinases (bla_OXA-48/48-like_ variants) and the *Klebsiella pneumoniae* carbapenemase (bla_KPC_).^2^ Intra- and interspecies horizontal transfer of these genes is facilitated by mobile genetic elements such as transposons and plasmids, resulting in rapid dissemination of carbapenem resistance.^3,4^

CPE have been isolated from human and animal gastrointestinal tracts, and from sewage, rivers and sink drains.^5–7^ Recent studies have highlighted that hospital wastewater sites may act as a CPE reservoir,^5^ but the diversity within this reservoir, genetic overlap with patient isolates, and likely directionality and rates of transmission remain unclear. Studies characterizing patient-to-patient transmission as the sole explanatory factor in dissemination have been unable to robustly explain most transmission events, suggesting that patient, staff and/or environmental reservoirs remain insufficiently considered.^8^ Few studies have evaluated within-niche diversity by sampling multiple isolates per individual and/or site.^9–11^

The Manchester University NHS Foundation Trust (MFT; UK) has experienced bla_KPC_-positive Enterobacterales (KPC-E) cases since 2009. Following a bla_KPC-2_-ST216-*Escherichia coli* outbreak on two cardiac wards and subsequent ward closure and plumbing infrastructure replacement, early environmental sampling after the ward reopened suggested rapid recolonization of wastewater sites with KPC-E, likely from transfers of colonized patients to the ward and ongoing evolution of new KPC-E strains in the environment.^12^ We therefore undertook a prospective study systematically sampling all wastewater sites on six wards for 6–12 months alongside patient rectal screening/clinical sampling, and used an anonymized electronic database to characterize patient admission, ward movement, sampling profiles and culture results. Multiple colonies from KPC-E-positive samples were sequenced to define within-niche diversity and consider modes of evolution and transmission.

## Patients and methods

### Study setting

MFT is a large centre in northwest England, UK, managing >10 000 patients per year. In response to the regional emergence of KPC-E,^13^ the Trust implemented an extensive Infection Prevention and Control (IPC) programme, consistent with UK guidelines.^14^ Despite this, in April 2015 there was a large *bla*_KPC_-*E. coli* outbreak in the cardiac unit,^12^ leading to its closure (September 2015 to January 2016) and complete refurbishment, including plumbing replacement. Subsequently, systematic wastewater site sampling was undertaken (see below).

To evaluate patient-level microbiological/admissions data, MFT electronic bacteriology records were linked, based on NHS numbers, to patient administration data, and anonymized. For this study, we analysed anonymized patient data from 1 January 2016 to 31 December 2016 inclusive, restricted to patients with exposure to any of six wards within the three study units (acute medicine, cardiology, geratology) during that time.

Patients and wastewater sites were considered as two distinct KPC-E reservoirs, with individual patients and specific environmental sites considered as niches.

### Environmental sampling and laboratory processing

All sink/drain/wastewater sites on the units/wards were sampled: the cardiac unit [wards 3 (W3)] and 4 (W4)]; the geratology unit [wards 45 (W45) and 46 (W46)]; and the acute medicine unit (wards AM1 and AM2) (Table S1, available as Supplementary data at *JAC-AMR* Online, shows sampling site designations/wastewater site types). All wastewater sites on both W3 and W4 were sampled fortnightly on rotation from 8 January 2016 to 28 December 2016. W45, W46, AM1 and AM2 wastewater sites were similarly sampled fortnightly, but from 18 July 2016 to 31 December 2016.

Environmental sampling was carried out by aspirating ∼20 mL of wastewater from sink P-traps, shower drains or toilets and performing enrichment-based culture (see Supplementary methods). Multiplex, real-time, quantitative PCR (for *bla*_NDM_, *bla*_KPC_, *bla*_OXA-48_)^12^ was performed on broths following incubation. A 10 μL sample of any *bla*_KPC_-positive-broth was streaked onto CPE-selective agar plates (Chromid CARBA) and reincubated (aerobically, 37°C).

### Patient sampling and laboratory processing

Routinely collected patient clinical samples were processed using standard operating procedures in line with UK Standards.^15^ In addition, a rectal CPE screening programme was in place from 2014 in line with national guidance (see Supplementary methods). Whereas all rectal swabs were directly screened for carbapenem resistance genes using the Cepheid Xpert Carba-R assay or an in-house multiplex PCR (identifying *bla*_KPC_, *bla*_NDM_ and *bla*_OXA-48_), clinical isolates were typically only profiled routinely in terms of carbapenem susceptibility. Species identification of isolates was performed by MALDI-TOF (Bruker Biotyper); antimicrobial susceptibility testing was performed as per EUCAST guidelines.^16^ To evaluate within-niche diversity in the environment and patients, up to five different CPE colonies were individually subcultured and stored for sequencing.

### DNA extraction and isolate sequencing

DNA was extracted from frozen subculture stocks with the QuickGene kit, as per the manufacturer’s instructions, with an additional mechanical lysis step post-chemical lysis (6 m/s at 40 s ×2 FastPrep; MPBio). Isolates were sequenced on the Illumina HiSeq 4000 generating 150 bp paired-end reads.

### Sequence data processing and analysis

Sequencing reads were processed;^17^ species identification was performed with Kraken2 (v2.0.8-beta; default settings),^18^ and if ≥50 sequenced isolates were of a single species, reads were mapped to species-specific references and variation called (see Supplementary methods). Recombination-adjusted phylogenies by species were created from core chromosomal single nucleotide variants (SNVs; padded to the reference genome length) using IQtree^19^ (v1.5.3; flags: -m GTR + G -blmin 0.00000001 -t PARS) and then ClonalFrameML^20^ (v1.0; default parameters). A threshold of ≤400 SNVs was used to define strain clusters (similar to Sheppard *et al*.;^21^ distribution of within-cluster SNV distances in Figure S1a–e for commonest species). For detailed sub-strain analysis for *K. pneumoniae* strain 9, sub-strains were defined on the basis of accessory genome clusters characterized using Panaroo (v1.2.9; default parameters including-clean-mode strict)^22^ and clustered using the R heatmap package.

Sequences were assembled using SPAdes (v3.6; default parameters).^23^ Multilocus sequence types were derived using BLASTn and species-specific databases (https://pubmlst.org). AMR genes were identified using ARIBA^24^ (v2.11.1) and the CARD database^25^ (version 2.0.0) and ISs using ISFinder (https://isfinder.biotoul.fr). *bla*_KPC_ genes, Tn*4401* transposons and the 5 bp target site sequences (TSSs) on either side of the Tn*4401* transposon reflecting transposition signatures were characterized using TETyper^26^ (version 1.1), a Tn*4401*b reference sequence, a 5 bp flank length and the default structural/SNV profiles. Arbitrary profile numbers were assigned based on the presence of unique profiles of AMR genes, plasmid replicons and ISs (Supplementary datasets 1 and 2).

For the analysis of *K. pneumoniae* strain 9 (see Results), putative transmission networks were inferred first using SCOTTI^27^ (2022–12-21) and then heuristically, linking isolates identified as clusters on the basis of accessory component profiles and geographic/temporal overlap (Supplementary methods).

### Statistics and data visualization

Descriptive statistics were calculated in R v3.6.2. For permutational analyses of variance, we used the ‘adonis’ function (vegan R package, v2.5-7)^28^ on a matrix of pairwise Gower distances based on a composite genetic profile of species-strain type, plasmid replicons, AMR genes, ISs and Tn*4401* + flanking TSSs, which was calculated using the ‘daisy’ function (R cluster package). To evaluate the impact of unit location or environmental site type on the odds of CPE positivity, we used logistic regression with robust standard errors clustered by environmental site sampled (R rms package, v6.2-0).^29^ For data visualization we used the ggplot2 R packages^30^ and BioRender (www.biorender.com).

### Data availability

Sequence data have been deposited in NCBI (BioProject accessions PRJNA768622, PRJNA514245; Supplementary dataset 2). We are not able to share the complete database of patient tests, results and admissions episodes; dates for patient admission/sampling are given as month/year only. Of note, labels represented in the figures and supplementary data are anonymized study-specific labels that are not patient-identifiable.

### Ethical considerations

This study was part of a Trust board-approved and UK Health Security Agency long-term outbreak response, and ethical approval was not required under NHS governance arrangements; all analyses were undertaken using anonymized data. This was reviewed by the Joint Research Office of the Oxford University Hospitals NHS Foundation Trust and the University of Oxford, who deemed that neither sponsorship nor research ethics review was required.

## Results

### Environmental sampling revealed high prevalence and clustering of wastewater KPC-E colonization

**Figure 1.**
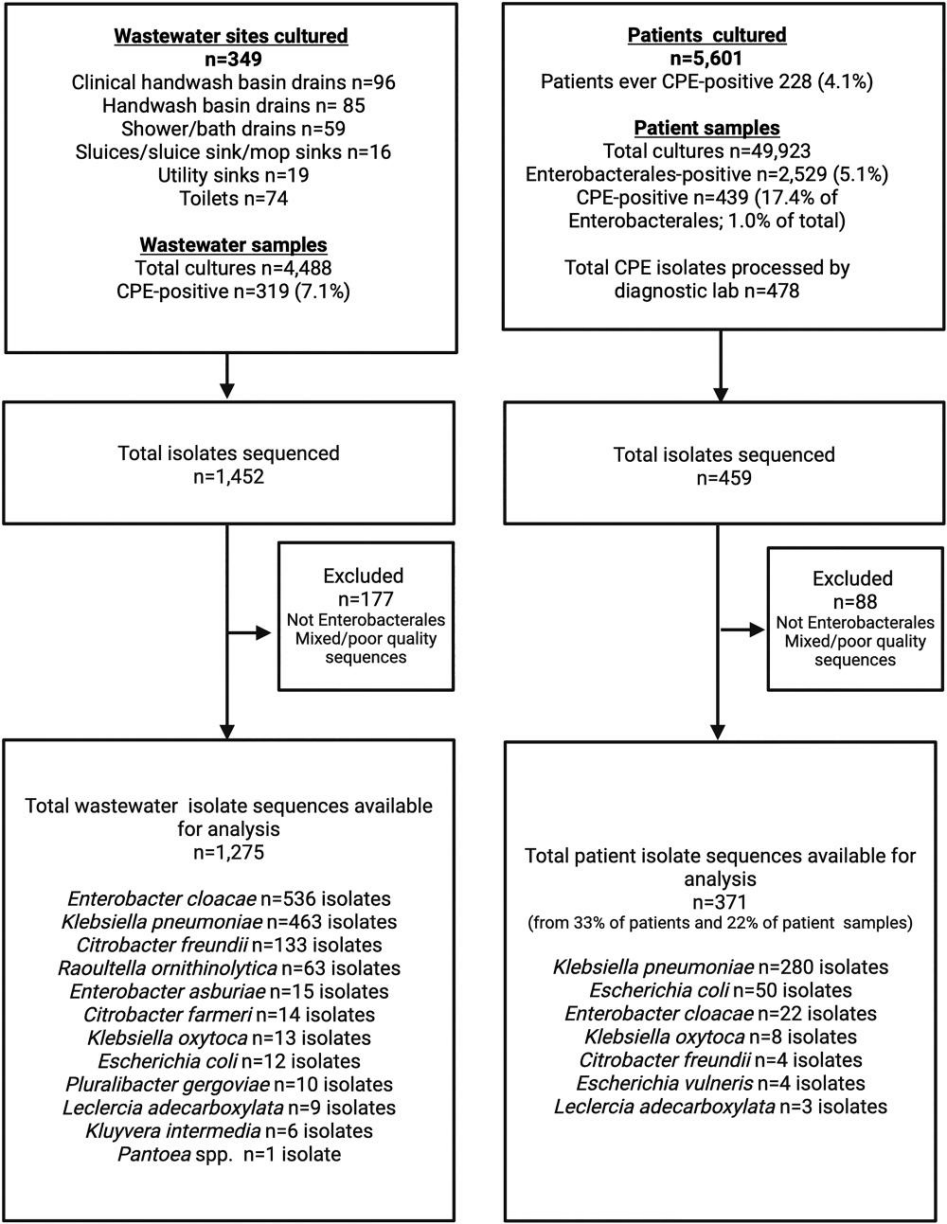
Sampling and sequencing flowchart for the study (created using www.biorender.com).

**Figure 2.**
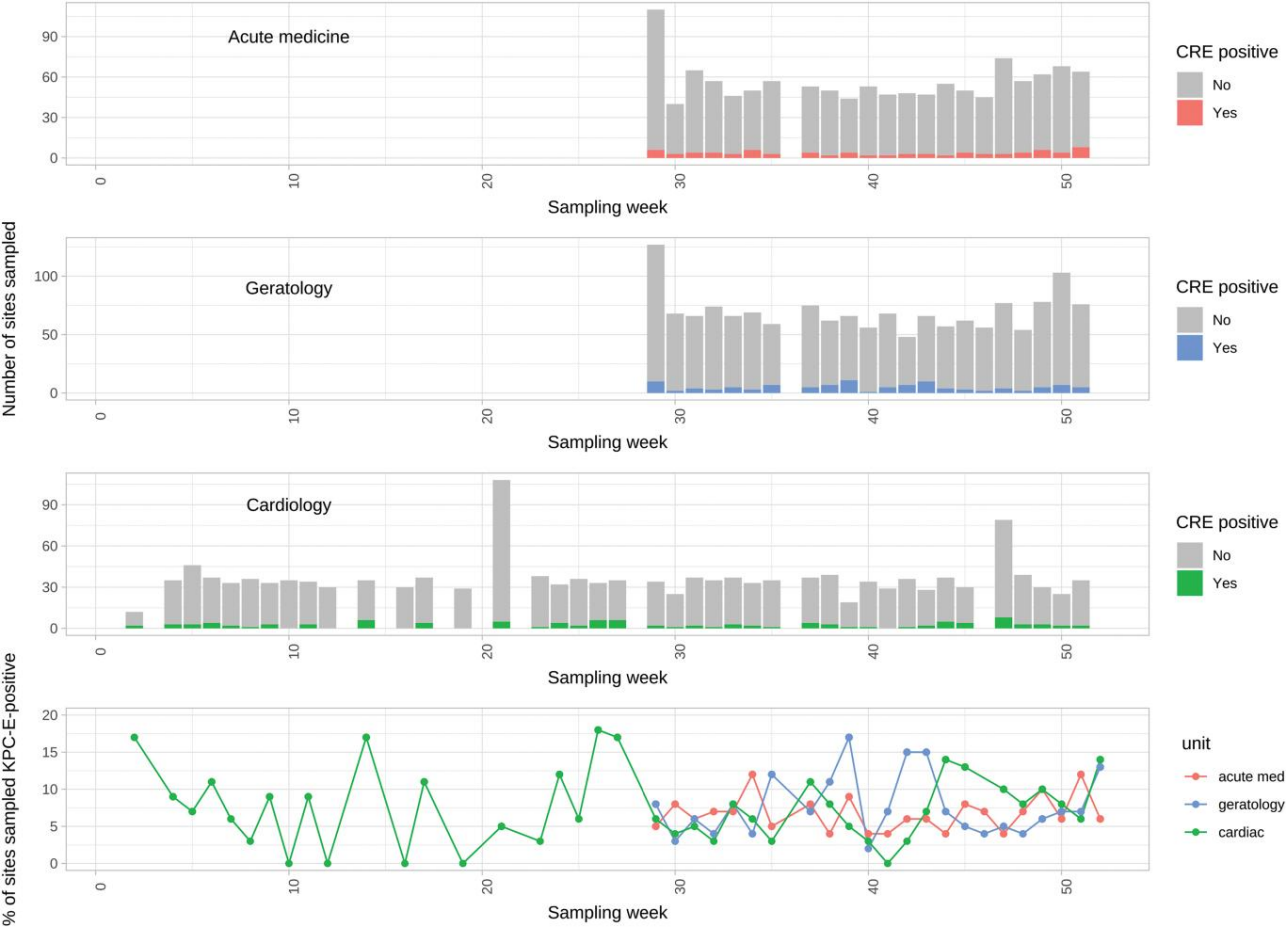
bla_KPC_-Enterobacterales (KPC-E)-positive environmental sites by Week in 2016 and hospital unit. The number of environmental site samples performed and KPC-E-positive samples by week of sampling, stratified by unit (top three panels), and the proportion of KPC-E-positive sites by week of sampling, again stratified by unit (bottom panel). Plumbing replacement on the cardiac unit (wards 3 and 4) was carried out at Week 0.

To characterize how densely and persistently ward wastewater sites were colonized with KPC-E over time, 349 sites across the six study wards were sampled a total of 4488 times over 6–12 months (Figure 1). We found that 101/349 (28.9%) sites and 319/4488 (7.1%) sampling events were KPC-E-positive, with no difference by unit (Figure 2, *P* = 0.908), or over time. Adjusting for environmental site type and unit location, shower/bath drain sites and sluices/sluice sinks/mop sinks were significantly more likely to be positive for KPC-E than others [adjusted OR (AOR) (95% CI): 2.69 (1.44–5.01), *P* = 0.0019; and AOR 2.60 (1.04–6.52), *P* = 0.041, respectively], and toilet water sampling less likely to be positive [AOR (95% CI): 0.28 (0.10–0.78), *P* = 0.015]; there was no evidence of a unit-dependent difference (Table 1). We found 7/349 (2.0%) sites to be persistently KPC-E positive (≥10 consecutive KPC-E-positive sampling events); interestingly five of these were medicines/treatment room handwash basin drains (of eight medicines/treatment room sinks in total) (Table S1, Figure S2).

**Table 1.** Association of environmental site type and unit location with bla_KPC_-Enterobacterales (KPC-E) positivity in environmental samples^a^

Environmental site	Number of sites	Number of sampling events KPC-E-positive (% of total sampling events)	Number of sampling events KPC-E-negative	OR (95% CI)	*P*
**Environmental site type**					
Clinical handwash basin drain	96	79 (5.9%)	1270	Reference	
Handwash basin drain	85	52 (4.6%)	1077	0.99 (0.51–1.93)	0.9735
Shower/bath drain	59	100 (19.2%)	421	2.69 (1.44–5.01)	**0**.**0019**
Sluice/sluice sink/mop sink	16	47 (17.9%)	215	2.60 (1.04–6.52)	**0**.**0410**
Toilet	74	5 (0.5%)	931	0.28 (0.10–0.78)	**0**.**0152**
Utility sink	19	36 (12.4%)	255	1.32 (0.47–3.68)	0.5965
**Environmental site location**					
Acute medicine unit	129	86 (6.6%)	1223	Reference	
Cardiac unit	76	111 (7.0%)	1453	1.07 (0.57–2.00)	0.8412
Geratology unit	144	122 (7.6%)	1493	0.88 (0.52–1.48)	0.6243

^a^Outputs of a multivariable logistic regression model with robust standard errors clustered by environmental site type and unit location; no other variables were considered in the model. *P* values <0.05 were considered significant and are highlighted in bold. No interactions with an interaction Wald *P* < 0.05 were observed.

### Approximately 1 in 25 patients were colonized or infected by KPC-E, with most cases reflecting rectal colonization

To determine the prevalence of infection in and asymptomatic colonization of patients with CPE, we analysed 49 923 culture results from 5601 patients. Of 2529 Enterobacterales-positive samples (2622 Enterobacterales isolates, 1247 patients), 439/2529 (17.4%) samples cultured at least one carbapenem non-susceptible Enterobacterales (*n* = 478 isolates in total) (Figures 1 and S3). For 344/439 (78.4%) of these isolates for which carbapenemase PCR data were also available, all contained at least one known carbapenemase gene, of which 303/344 (88.0%) were bla_KPC_ (Table S2). CPEs were cultured from 228 patients (4.1% of all patients cultured, 18.2% of all patients with an Enterobacterales-positive culture) during the study period (median = 1; IQR: 1–2; range: 1–17 CPE isolates/patient).

Most CPE-positive isolates came from rectal screens [*n* = 387/478 (81.0%)]; others came from non-screening clinical specimens [91/478 (19.0%)] (Table S3). Most CPE-positive patients [178/228 (78%)] were CPE-positive only on rectal screen, but 23/228 (10%) were positive only on clinical culture, and 27 (12%) on both. By routine laboratory methods, >20 species and eight genera were represented amongst the CPEs, with *K. pneumoniae*, *E. coli* and *Enterobacter cloacae* complex predominating [434/478 (90.8%)]; notably, the proportions of carbapenem-resistant isolates within these three species varied [*n* = 284/593 (48%) versus 106/1404 (8%) versus 44/110 (40%), respectively; Fisher exact test, *P* < 0.001, Figure S3].

**Figure 3.**
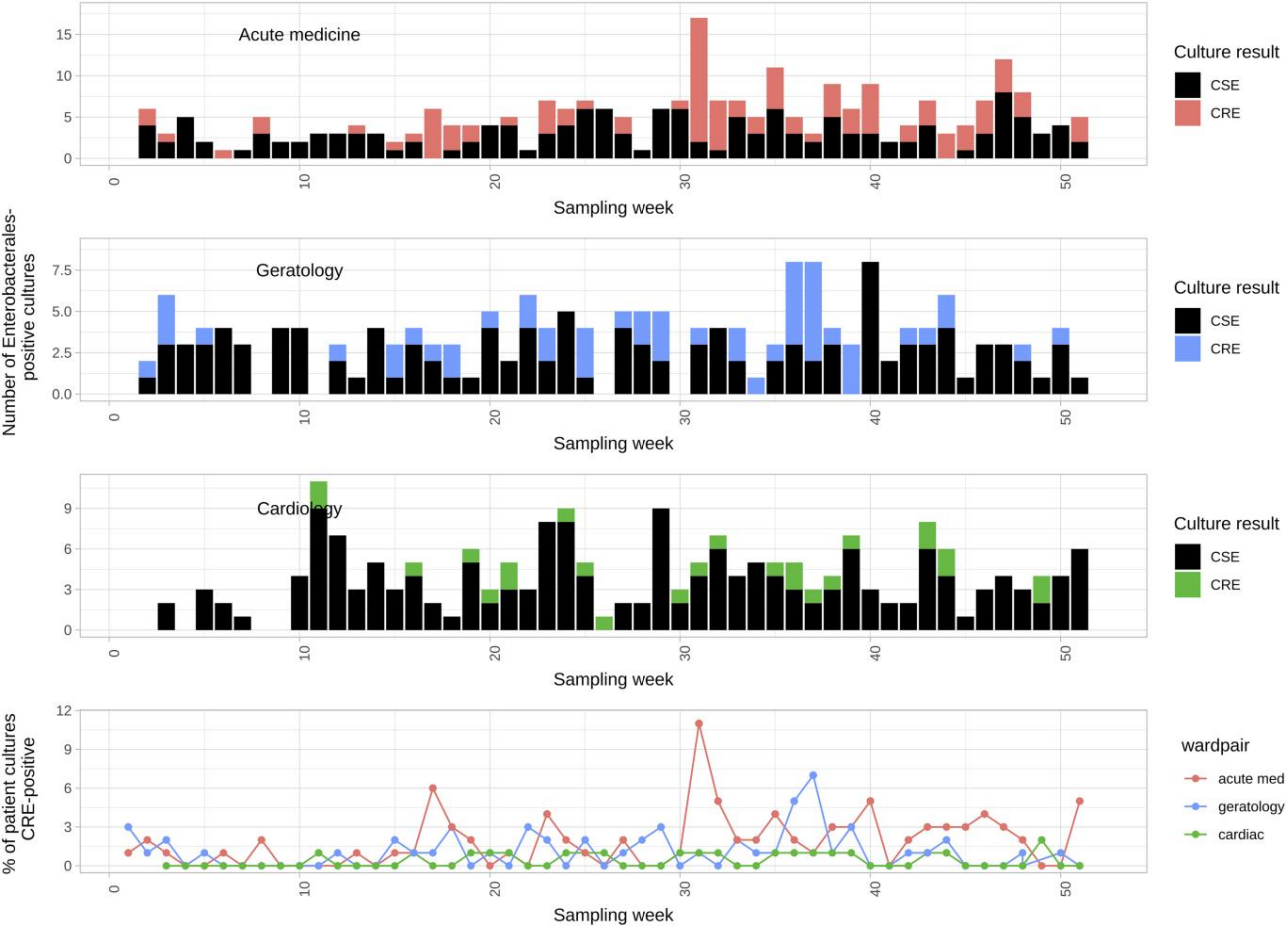
Carbapenem-susceptible Enterobacterales (CSE) and carbapenem-resistant-Enterobacterales (CRE) patient cultures by week in 2016 and hospital unit. Top three panels show counts of Enterobacterales culture-positive samples stratified by unit and carbapenem susceptibility; bottom panel shows the proportion of all cultures taken that were CRE, again stratified by unit.

The proportion of patient CPE-positive cultures was lower than amongst environmental samples, typically ∼3%–4% (Figure 3, bottom panel). Occasional peaks in prevalence were observed, consistent with outbreaks (e.g. Week 31, acute medicine unit). CPE prevalence amongst patient cultures on the refurbished cardiology unit was 0% (97.5% CI: 0–0.02) for the first 10 weeks post-reopening, although two utility room sink drains were positive for KPC-E a day after W3 reopened to patients (11 January 2016). After Week 20 the unit was clearly recolonized (Figure 2), with positive environmental CPE-cultures observed in 26/27 (96%) weeks of sampling and positive patient CPE-cultures observed in 16/32 (50%) weeks of sampling.

CPE culture-positivity was therefore approximately seven times higher in wastewater sites (28.9%) sampled than in patients sampled throughout the study period, and just over three-quarters of patient CPE culture-positives reflected carriage (i.e. positive on rectal screens), highlighting these as major potential reservoirs for transmission. Plumbing replacement on the cardiac unit had only a very transient effect on environmental and patient CPE culture-positivity.

### Structuring of KPC-E diversity by patient and environmental niche, with most diversity explained by the individual niche

**Figure 4.**
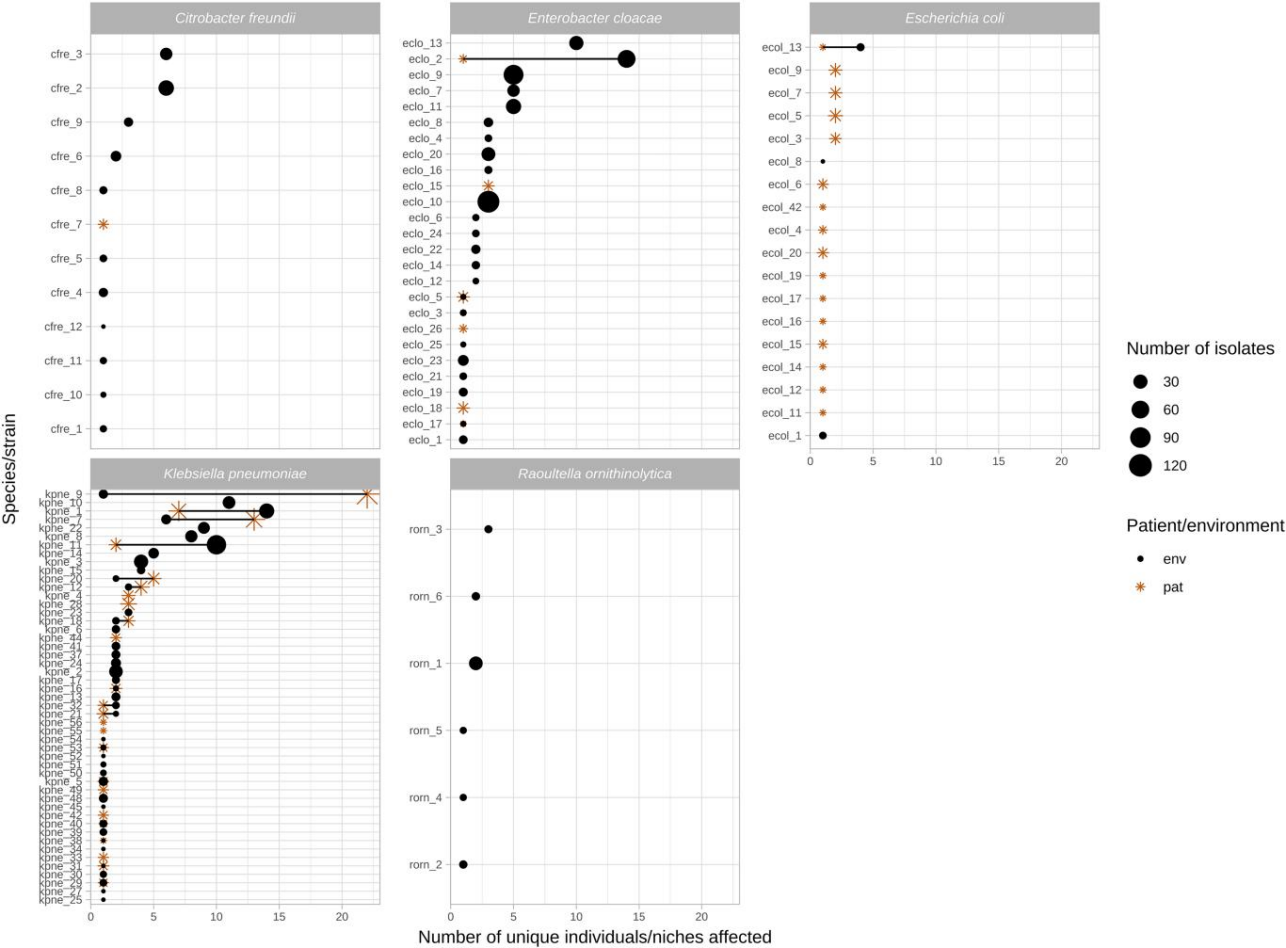
Number of unique patient/environmental niches colonized by common *bla*_KPC-2_ Enterobacterales species/strains and the total number of isolates for each cluster. Includes species/strains if >50 isolates of a species were identified (see Methods). Stars denote patient niches, circles wastewater niches; larger shape size denotes a larger number of isolates sequenced. Strains observed in both niches are either overlapping shapes (same number of niches), or joined by a line (different numbers of niches).

Given that culture-based microbiology cannot characterize genetic diversity within species, and this diversity is relevant to understanding carbapenem gene dissemination and transmission, we undertook genome sequencing of isolates. We successfully sequenced 1646 CPE isolates (1275 environmental, 371 patient isolates; Figure 1 and Supplementary dataset 2); all contained bla_KPC-2_, and 13 KPC-E species were identified across patients and environmental reservoirs. Amongst five species with ≥50 sequenced isolates for which phylogenetic analysis and clustering was performed, 109 unique strains were represented. Of the 109 strains, 61 (56%) were found only in the environment, 27 (24%) only in patients, and 21 (19%) in both patients and the environment (Figure 4); the median strain cluster size was five isolates (range: 1–122), and the median number of unique niches (i.e. unique patients or wastewater sites) affected by a strain was two (range: 1–23).

To identify the diversity of mobile genetic elements that could support horizontal gene transfer (HGT), we evaluated plasmid replicon and IS profiles in isolates. We also defined Tn*4401* TSS diversity because Tn*4401* mobilizes *bla*_KPC_ and TSS diversity can be a marker of transposition frequency. Amongst sequenced isolates, 100 plasmid replicon profiles, 754 IS profiles, 939 AMR gene profiles and 70 Tn*4401*/TSS types were identified (Supplementary dataset 2). More Tn*4401*/TSS types were observed in the environment versus patients [66/70 (94%) of types versus 12/70 (17%), respectively; Fisher exact test, *P* < 0.001], and all the isolates with more than one set of TSSs were seen in environmental sites, consistent with these representing a more favourable reservoir for Tn*4401*-associated transposition/evolution and dissemination.

For colonized niches within patient or environmental reservoirs, using a metric of genomic diversity based on strain, plasmid replicon profile, AMR gene profile, IS profile, and Tn*4401*+5 bp TSS profiles, most variance was explained by the specific individual niche (i.e. patient or environmental site) sampled (*R*^2 ^= 50.5%; *P* < 0.001), versus small but significant contributions made by reservoir type (i.e. patient versus environmental reservoir; *R*^2 ^= 5.6%; *P* < 0.001) and unit location (*R*^2 ^= 2.8%; *P* < 0.001).

For the 15 cases where exactly the same strain, AMR gene profile, IS profile, plasmid profile and Tn*4401*/TSS type were seen in patient and environmental niches (Figure S4), and considering only event-pairs where chromosomal SNP distances were within ≤5 SNPs and there was a change in sampling site, the temporal relationship of these supported patient-to-environment transmission in 13/29 (45%) events, environment-to-patient transmission in 2/29 (7%) events, environment-to-environment transmission in 1/29 (3%) events, and patient-to-patient transmission in 13/29 (45%) events (test of equality of proportions, *P* < 0.001).

### Environmental and patient niches harboured diverse KPC-E within single samples and over time

**Figure 5.**
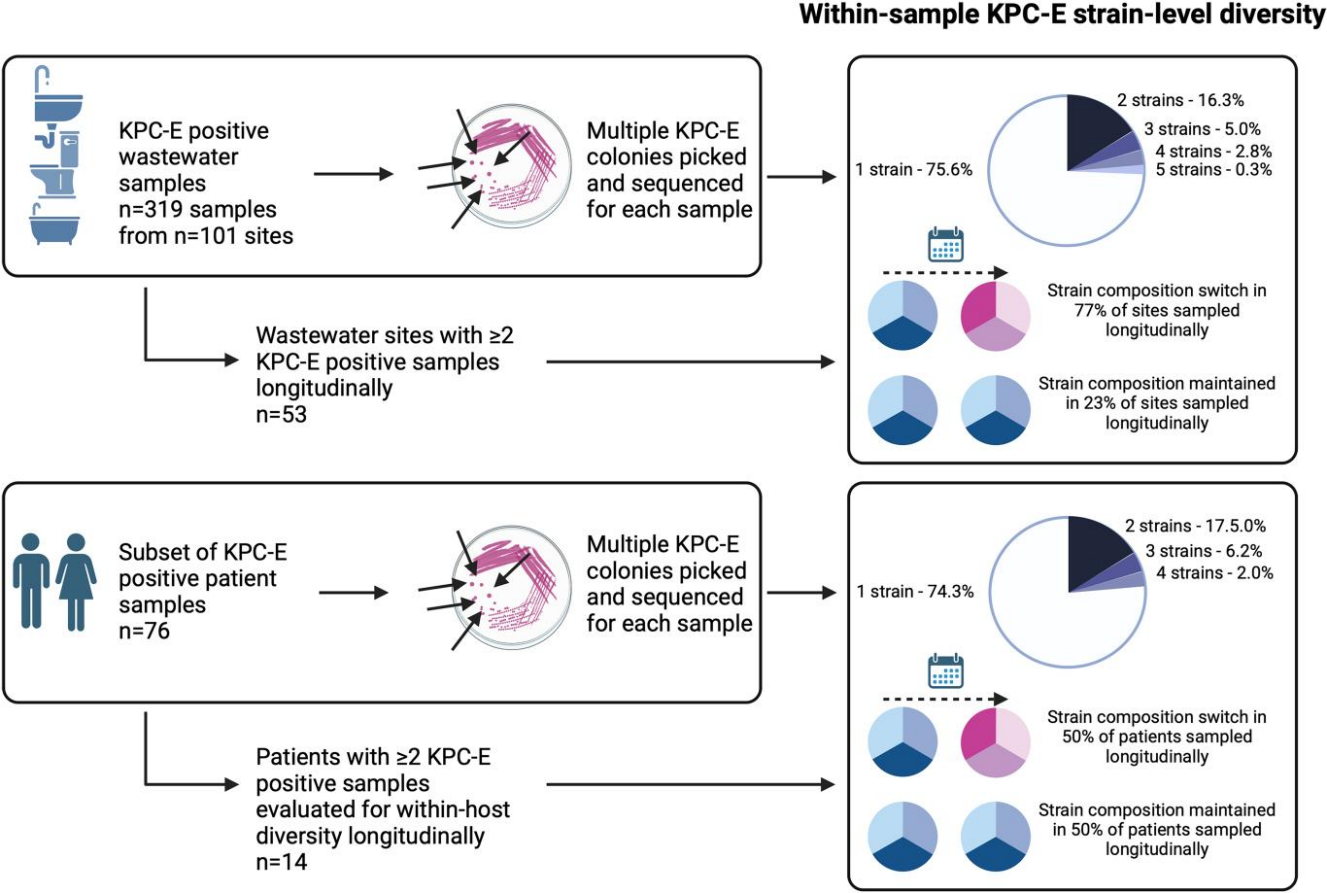
Schematic of within-reservoir and KPC-E sample diversity using genomics (generated in www.biorender.com).

Environmental sites showed evidence of major strain-level diversity: from the 101/349 sites from which bla_KPC-2_-Enterobacterales isolates were successfully sequenced at any timepoint, 78/319 (24.4%) KPC-E-positive samples had more than one bla_KPC-2_-Enterobacterales strain, namely 52/319 (16.3%) with two strains, 16/319 (5.0%) with three, 9/319 (2.8%) with four and 1/319 (0.3%) with five strains (Figures 5 and S5). Over the study period, colonized environmental sites had a median of three (range: 1–17, IQR: 2–5) bla_KPC-2_-Enterobacterales strains; 49/101 sites (49%) were apparently colonized by a single bla_KPC-2_-Enterobacterales strain only, of which 38 were at single sampling-points, and 11 sites were positive with the same strain at two or more timepoints.

For patient samples, based on routine laboratory microbiology, evidence of mixed-species KPC-E colonization/infection was found in 35/439 (8%) samples from 26/228 (11%) patients; these were mostly *E. coli*/*K. pneumoniae* mixtures (*n* = 17 samples; maximum two species identified). Based on a genomic evaluation of 457 colonies from 97 samples from 76 patients, 25/97 samples (26%) had more than one strain per sample, namely 17/97 samples (18%) with two PKC-E strains, 6/97 (6%) with three strains and 2/97 (2%) with four strains (Figures 5 and S5)—i.e. considerably more diversity identified than based on routine laboratory species identification data (26% versus 8% samples; Fisher exact test, *P* < 0.001).

For a subset of 14 patients and 53 wastewater sites where within-sample diversity was evaluated by sequencing and two or more longitudinal samples were positive, longitudinal switches in KPC-E species-strain composition were common, occurring in 7/14 (50%) of patients and 41/53 (77%) wastewater sites (Figures 5 and S5).

### Genomic analysis revealed distinct population biology for different bla_KPC-2_-Enterobacterales strains

To investigate different modes of bla_KPC-2_ dissemination, we characterized three sequenced KPC-E strain clusters, including the largest environmental-only cluster (*E. cloacae* strain 10; *n* = 122 isolates), a large cluster involving patients and the environment (*K. pneumoniae* strain 9; *n* = 106 isolates) and a cluster in which signatures of Tn*4401* transposition appeared highly frequent (*K. pneumoniae* strain 11; *n* = 96 isolates).

#### Scenario 1: dynamic changes in AMR gene, IS, plasmid replicon and Tn4401 profiles in E. cloacae strain 10 in sink drains

**Figure 6.**
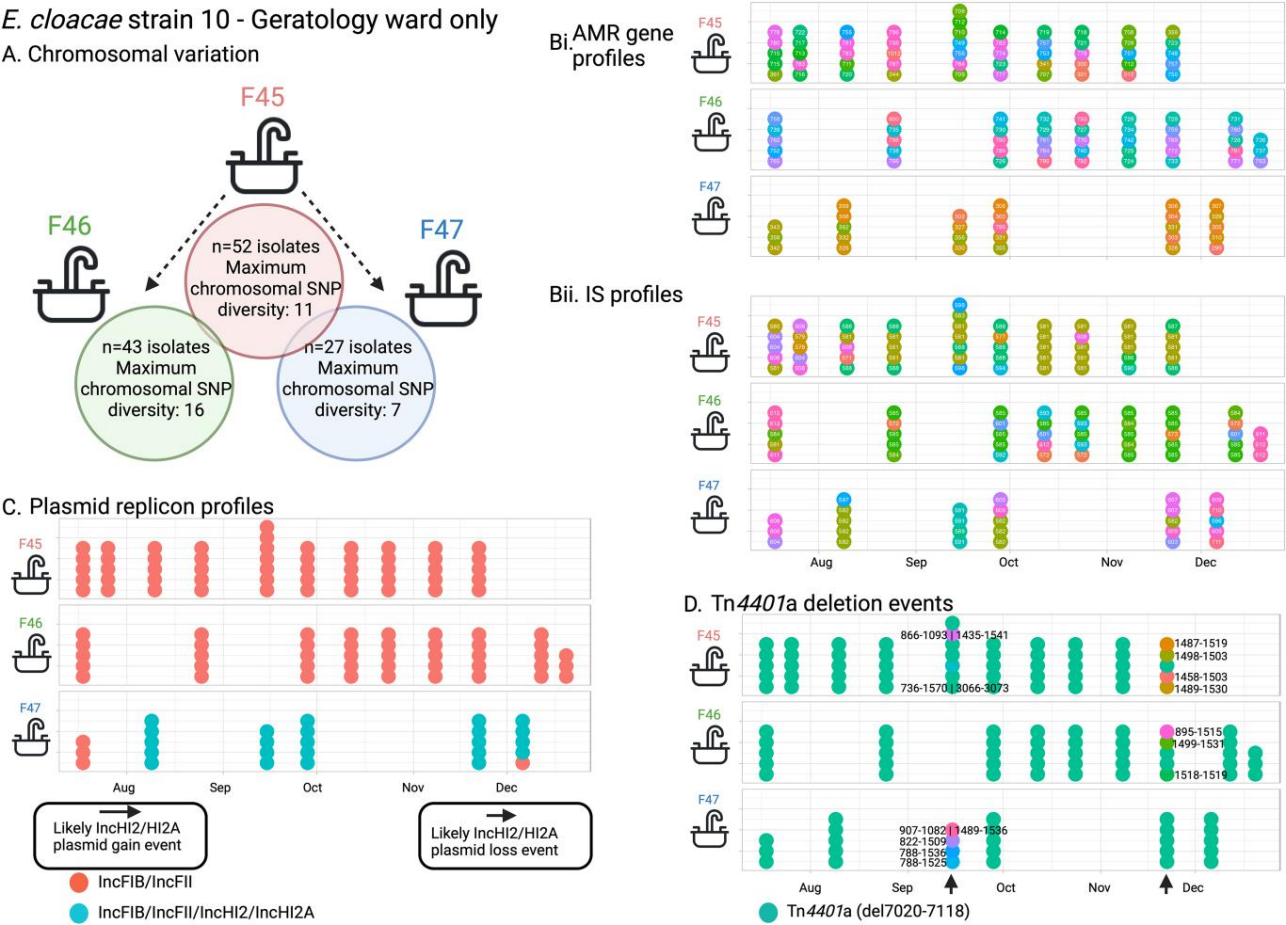
*Enterobacter cloacae* strain 10: features of population structure and transmission dynamics—niche restriction, persistence and genetic turnover. Summaries of (a) chromosomal SNP distances for *E. cloacae* strain 10 across the three sites it colonized; (bi) AMR and (bii) IS profiles; (c) plasmid replicon profiles; and (d) deletions in Tn*4401*a in isolates. Each sequenced isolate is plotted as a dot reflecting within-sample diversity at given timepoints and longitudinally, with colour in panels (bi) and b(ii) reflecting distinct AMR and IS profiles respectively and numbers within the dots representing the numeric identifier for profiles listed in Supplementary dataset 1.

*E. cloacae* strain 10 (eclo10, ST32) was a site-restricted but persistent colonizer of three co-located sink drains on a geratology ward [in a drug room clinical handwash basin drain (F45), utility sink drain (F46), and a separate treatment room clinical handwash basin drain (F47)] over ∼5 months (18 July 2016 to 20 December 2016). At the core chromosomal level, strains appeared largely structured by site, with 7 SNVs separating the majority of isolates in F45 from those in F47, and 14 SNVs those in F46 from F45. Three isolates in F47 and one isolate in F46 clustered with the F45-associated isolates; sampling dates suggest that these represented transmission events from F45 (Figure 6a). There was evidence of rapid churn in AMR gene and IS content (111 and 36 different profiles respectively; Figure 6bi and 6bii) in the relatively stable strain background, and almost all isolates in F47 had acquired an additional set of IncHI2/HI2A replicons on a background of FIB/FII, consistent with plasmid gain/loss (Figure 6c). Although the Tn*4401* type and flanking sequences were largely identical [Tn*4401*a-ATTGA-ATTGA in 105/122 (86%) isolates across sinks], there was evidence of unique additional Tn*4401* deletions occurring in multiple isolates at several distinct timepoints in the three sink sites (Figure 6).

#### Scenario 2: rapid clonal patient–patient transmission of K. pneumoniae strain 9

**Figure 7.**
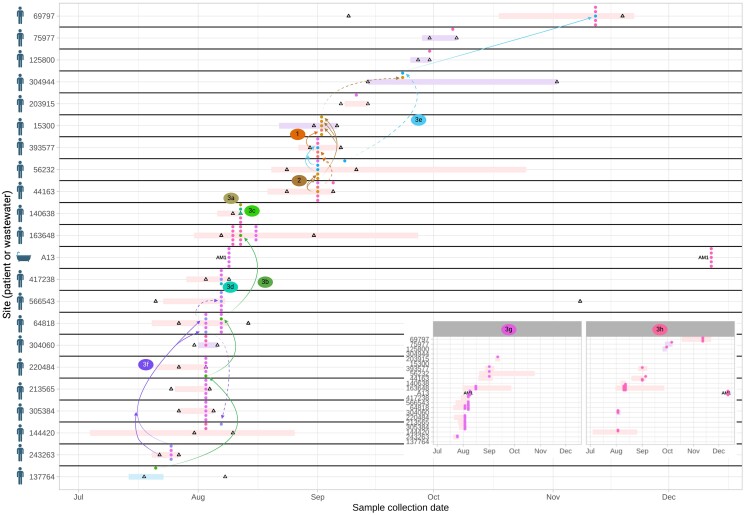
*Klebsiella pneumoniae* strain 9: features of population structure and transmission dynamics—rapid patient-patient transmission. Putative transmission network for sub-lineages of *K. pneumoniae* strain 9, defined by accessory genome clustering (clusters 1, 2, 3a–h). Horizontal bars represent admission episodes to study wards for each patient (pale blue = W45, geratology; pale pink = AM1, acute medicine; pale purple = AM2, acute medicine; A13 represents the only environmental (shower) site involved. Empty black triangles denote CPE-negative rectal screens on patients. Arrows denote possible transmission links (dashed arrows denote events that have no geographical overlap or could be associated with two links based on negative screen, geographical location and timing). For clarity, arrows are not drawn for clusters 3g and 3h, which affected the most patients—these are shown in detail as an inset panel at the bottom right. In some cases patients were colonized by other species-strains contemporaneously (not shown here). (Figure generated in ggplot and www.biorender.com).

*K. pneumoniae* strain 9 (kpne9, ST252) was highly related (Figure S6; maximum six SNVs across strain, 68 isolates with zero SNVs between them), and spread among 21 patients across three wards on two units (acute medicine AM1 and AM2, and geratology W46), with a dense outbreak in August 2016, most consistent with direct/indirect patient–patient transmission (Figure 7). bla_KPC-2_ was consistently nested in Tn*4401*-ATTGA-ATTGA, except for one isolate, which acquired a Tn*4401*-associated mutation (isolate: 2216698_14, C4620T), and another in which the right TSS could not be identified (isolate: 2035791_11). Among the 106 isolates there was evidence of four transient plasmid replicon acquisitions on a background of the stable presence of a set of IncFIB/IncFII replicons (Figure S6). Transmission probabilities inferred by SCOTTI were weak (see Supplementary methods and Figure S7), as seen in other studies,^8^ but using accessory cluster profiling to further discriminate amongst strains suggested that there were several discrete transmission clusters (Figure S8), with some individuals involved in several of these (i.e. harbouring multiple distinct sub-lineages simultaneously, Figure 7). A single shower drain site was involved (A13), which was likely a seeded bystander from affected patients, but may have contributed to transmission of sub-strain 3g (Figure 7).

#### Scenario 3: cross-unit dissemination with evidence of substantial Tn4401-associated bla_KPC_ transposition in K. pneumoniae strain 11

**Figure 8.**
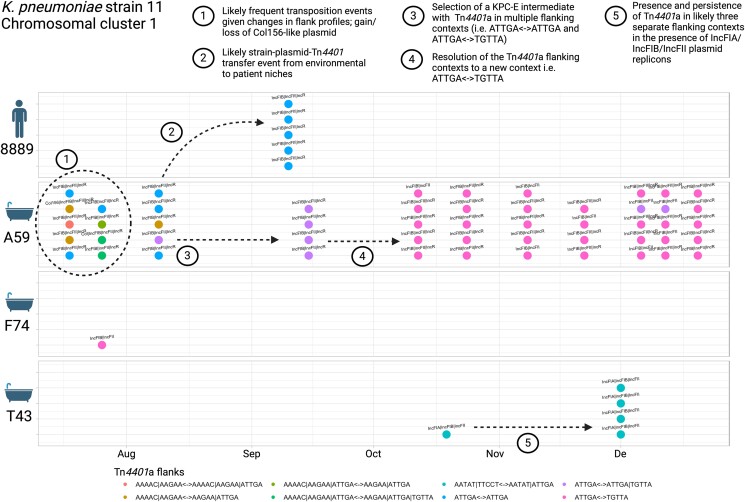
*Klebsiella pneumoniae* strain 11 cluster 1: features of population structure and transmission dynamics—multiple Tn*4401* transposition events. Examples of possible transmission/genetic events are annotated on the figure. Each isolate sequenced is represented as a dot, with colour denoting the TSS profiles observed. Plasmid replicon profiles are annotated as text. (Figure generated in ggplot and www.biorender.com).

Given that Tn*4401* transposition is associated with bla_KPC_ dissemination,^21^ we also evaluated whether the number of different Tn*4401*-TSS types was different across strains, noting that the observed frequency of different Tn*4401*-TSS combinations was higher in *K. pneumoniae* strain 11 (kpne11, ST11) than in other strains (Figure S9). On further analysis, kpne11 represented two sub-clusters, separated by ∼40 SNVs (Figure 8, designated ‘cluster 1’ and ‘cluster 2’; Figure S10), both of which were isolated from patients and wastewater sites across ward settings, including the cardiac unit after plumbing replacement. Within both clusters of isolates there were 10 different Tn*4401*a flanking signatures observed, only 1 of which was seen in the other >1600 isolates sequenced in the study (Tn*4401*a-1_ATTGA_ATTGA, the most common flanking signature observed overall), and therefore most strongly consistent with the occurrence of an atypical and increased number of transposition events within this *K. pneumoniae* strain. Although all isolates contained IncFIB and IncFII plasmid replicons, evidence of plasmid gain/loss events as demonstrated by changes in plasmid replicon profiles also appeared common (Figure 8 for cluster 1).

## Discussion

This work highlights the substantial KPC-E genetic diversity and flux in a *bla*_KPC_ genetic context and in niches that can occur in both human and wastewater reservoirs in healthcare settings over short timeframes. KPC-E dissemination dynamics appeared highly variable, making a generalized framework of transmission, evolution and bla_KPC_ sharing among these species very challenging. Although structuring at the strain level was observed by niche and species (Figure 4), likely transmissions occurred in a non-negligible manner in all directions from patients to other patients and the environment, and from the environment to patients and other environmental sites. The environmental reservoirs and certain species-strain contexts (such as *K. pneumoniae* strain 11) appeared to amplify bla_KPC-2_ transposition events.

Although some environmental sites were persistently colonized with individual strains over months, many appeared to be only transiently colonized. Sites particularly associated with KPC-E colonization included sluices, sluice sinks, mop sinks and shower/bath drains, as supported by other studies,^5^ and consistent with these representing a major interface between human faeces and premise plumbing. Toilets were less likely to be KPC-E-positive, perhaps because of regular flushing or particular cleaning protocols; consistent with this, several studies have demonstrated that daily bleach application decontaminates these sites.^31,32^

Our study underscores the detailed and dense sampling effort required to understand tracking AMR gene outbreaks across reservoirs, because so much diversity in genetic contexts can be observed as in this study, and this can fluctuate rapidly. This problem has also been demonstrated in other recent work investigating bla_OXA-48_, where only patient-based sampling was undertaken and most bla_OXA-48_-associated transmission events were non-delineated.^8^ Our study clearly shows that different species and lineages represent different epidemiological risks and have their own behaviours, and a focus on single, clonal strains as mediators of AMR gene transmission becomes less relevant. However, targeting large numbers of single isolates for sequencing is time consuming and resource intensive. One strategy might be to use long-read metagenomics, enabling reconstruction of genetic contexts around AMR genes, or long-read metagenomics of cultured sub-populations of isolates grown on relevant selective media to avoid wasted sequencing effort; this would require further evaluation.

The importance of evaluating asymptomatic carriage, characterizing within-host diversity of strains, considering rapid acquisition/loss of strains over short timeframes, and including environmental sites in transmission networks has been partly demonstrated in previous studies investigating ESBL *E. coli*^10,33^ and *Enterococcus* spp.,^34^ but generally these features are not jointly investigated because this is resource-intensive and analysis is challenging. However, our study highlights that without this effort, such as in studies that consider only single or infrequent sampling timepoints, and do not investigate asymptomatic colonization or consider within-site diversity,^35^ relevant transmission events may easily be missed, leading to incorrect assumptions about transmission networks and therefore potentially ineffective interventions.

There are several limitations to this work. We only evaluated a subset of CPE-positive patients and their samples using genomics (33% and 22%, respectively); we anticipate many transmission events were missed. We were not resourced to perform long-read sequencing to enable plasmid sequence reconstruction, and short-read-based profiling is relatively low-resolution and can be misleading;^21^ we therefore avoided a detailed plasmid analysis, which would be important future work. However, the diversity we observed in genetic contexts supporting *bla*_KPC_ was substantial, consistent with high rates of transfer. A further limitation was the absence of metadata capturing relevant selection pressures in each niche, including cleaning protocols for environmental reservoirs and drug prescription data for patients. We focused only on Enterobacterales and may have missed HGT of carbapenemase genes to other bacteria. Our approaches to evaluating some of the genetic changes and transmission events observed were heuristic and manual, partly because the data were highly heterogeneous and complex. The study sampling was undertaken several years ago in 2016–2017, but similar polyspecies, polyclonal carbapenemase gene outbreaks associated with hospital wastewater sites continue to be described,^36^ and as such we believe the findings make an important contribution to our understanding of the microbial ecology of the hospital built environment.

Further optimization of methods to evaluate multilevel genetic transmission and within-sample diversity without individual colony-level characterization is needed. Holistic approaches using fully reconstructed chromosome/plasmid assemblies to quantify the diverse types of genomic transmission (including clonal transmission, horizontal transfer at the gene, transposon and plasmid levels) are also required to better understand what facilitates emergence, selection and persistence of different lineages and genetic vectors of bla_KPC_. This information will improve our understanding of how to intervene to limit the transmission of drug-resistant Enterobacterales in healthcare settings.

## Supplementary Material

dlae140_Supplementary_Data

## Supplementary data

Figures S1 to S10, Tables S1 to S3, Supplementary methods and Supplementary datasets are available as Supplementary data at *JAC-AMR* Online.
